# Charge Localization Induced by Pentagons on Ge(110)

**DOI:** 10.1021/acs.jpcc.2c06399

**Published:** 2022-12-28

**Authors:** Dennis J. Klaassen, Carolien Castenmiller, Harold J.W. Zandvliet, Pantelis Bampoulis

**Affiliations:** Physics of Interfaces and Nanomaterials, MESA+ Institute, University of Twente, P.O. Box 217, 7500AE Enschede, Overijssel, The Netherlands

## Abstract

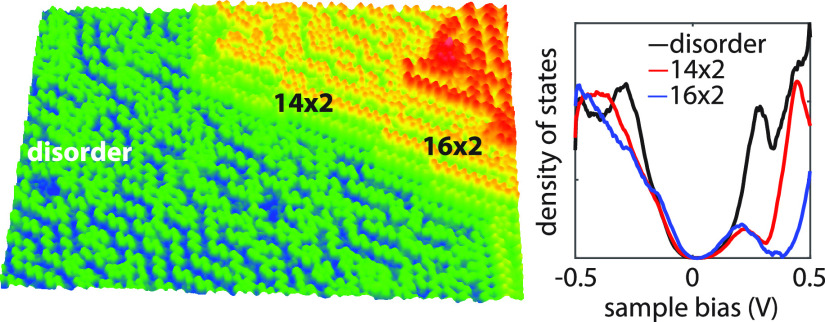

The Ge(110) surface reconstructs into ordered and disordered phases, in which the basic unit is a five-membered ring of Ge atoms (pentagon). The variety of surface reconstructions leads to a rich electronic density of states with several surface states. Using scanning tunneling microscopy and spectroscopy, we have identified the exact origins of these surface states and linked them to either the Ge pentagons or the underlying Ge–Ge bonds. We show that even moderate fluctuations in the positions of the Ge pentagonal units induce large variations in the local density of states. The local density of states modulates in a precise manner, following the geometrical constraints on tiling Ge pentagons. These geometry-correlated electronic states offer a vast configurational landscape that could provide new opportunities in data storage and computing applications.

## Introduction

As material dimensions approach nanometer length scales, quantum confinement^1^ and reduced dielectric screening^2^ at disordered states dominate the material’s (opto-)electronic and magnetic properties.^3^ Understanding the origin and impact of disorder is thus critical in unlocking the potential of materials in technological applications. Among the traditional low-index Si and Ge surfaces, the (110) surface has a relatively high surface free energy.^4−11^ These surfaces have gained attention due to developments in FinFET technologies.^12,13^ However, the high surface-free energy of the (110) surfaces leads to faceting and surface reconstructions. The types of reconstructions depend on several parameters during preparation, including annealing temperature and cooling rate. Often, the reconstructions do not fully form, resulting in disordered regions.^4^ Owing to this complexity, the surface structure of the reconstructed Ge(110) have not yet been fully understood. Understanding local structural order and disorder on semiconductors is important for a number of processes, such as the growth of metal nanostructures^14−18^ as well as the influence of the surface disorder on the material’s electronic properties.^19,20^ This is important because due to disorder, charge carriers can become strongly localized, influencing the local density of states and the material’s response to external stimuli, such as strain, electric field, adsorbates, etc.^21−23^ For example, when an electric field is applied in strongly localized electron systems, charge carriers hop between localized states from one randomly scattered location (e.g., dopant atom) to the other,^24−29^ resulting in non-linear responses and high resistivities.

Although disorder might reduce the usefulness of materials for some applications, it can be sometimes beneficial.^30^ The disordered component is often not random, but follows specific correlations or patterns.^23,31−33^ It reflects bonding arrangements, molecular orientations, charge states, atom displacements, defects, impurities, chemical composition, and magnetic structure.^30,34^ Understanding and manipulating disorder could provide the means toward new functionalities of conventional semiconductor crystals, offering a vast and ever-expanding configurational landscape.^31^ This combinatorial flexibility of disordered semiconducting crystals may offer a unique opportunity for data storage and new forms of computing,^31,35^ such as quantum and neuromorphic computing.^36−40^ These considerations have driven decades of in-depth studies to either minimize and circumvent or understand, regulate, and exploit disorder.^28,31,41,42^ According to Simonov and Goodwin,^31^ a challenge remains in the experimental investigation and characterization of disorder in materials and its impact on the material’s properties and specific functionalities.

Here, we use comprehensive scanning tunneling microscopy and spectroscopy (STM, STS) to experimentally investigate correlations in ordered and disordered reconstructions on the Ge(110) surface. Using spatially resolved d*I*(*V*)/d*V* mapping of the Ge(110) surface, we have identified the origins of several surface states on both ordered and disordered domains. These electronic states are strongly localized following precisely the geometry of the Ge pentagons (the basic unit of all the (110) reconstructions). Fluctuations in the positions of the Ge pentagons induce large variations in the local density of states, making it a dominant source of electronic inhomogeneity that correlates well with the position and density of the Ge pentagons.

## Experimental Methods

Scanning tunneling microscopy and spectroscopy were performed at 77 K (in a bath cryostat filled with liquid nitrogen) with Pt/PtIr tips, in an ultrahigh vacuum system (Omicron LT-STM) with a base pressure of 3 × 10^–11^ mbar. The Ge(110) samples were cut from nominal flat, single-side polished, and nearly intrinsic (50–60 Ω cm) Ge wafers. Before inserting the sample into the ultrahigh vacuum chamber, they were thoroughly cleaned in isopropanol alcohol. Thereafter, the Ge(110) samples were outgassed overnight at about 800 K. Subsequently, we cleaned the samples by several cycles (7–8) of argon ion bombardment followed by annealing at temperatures of 1100 (±25) K. This procedure results in atomically clean and defect-free reconstructed Ge surfaces.^4,43^ The relative occupation of the various reconstructions can be tuned by varying the cooling time after a high-temperature anneal. A fast cooling rate leads to an increase in the disordered domains. Since we aim at a detailed study of the disordered domains, we have rapidly cooled down our samples. Differential conductance mapping and d*I*(*V*)/d*V* curves were obtained using a lock-in amplifier.

## Results and Discussion

**Figure 1:**
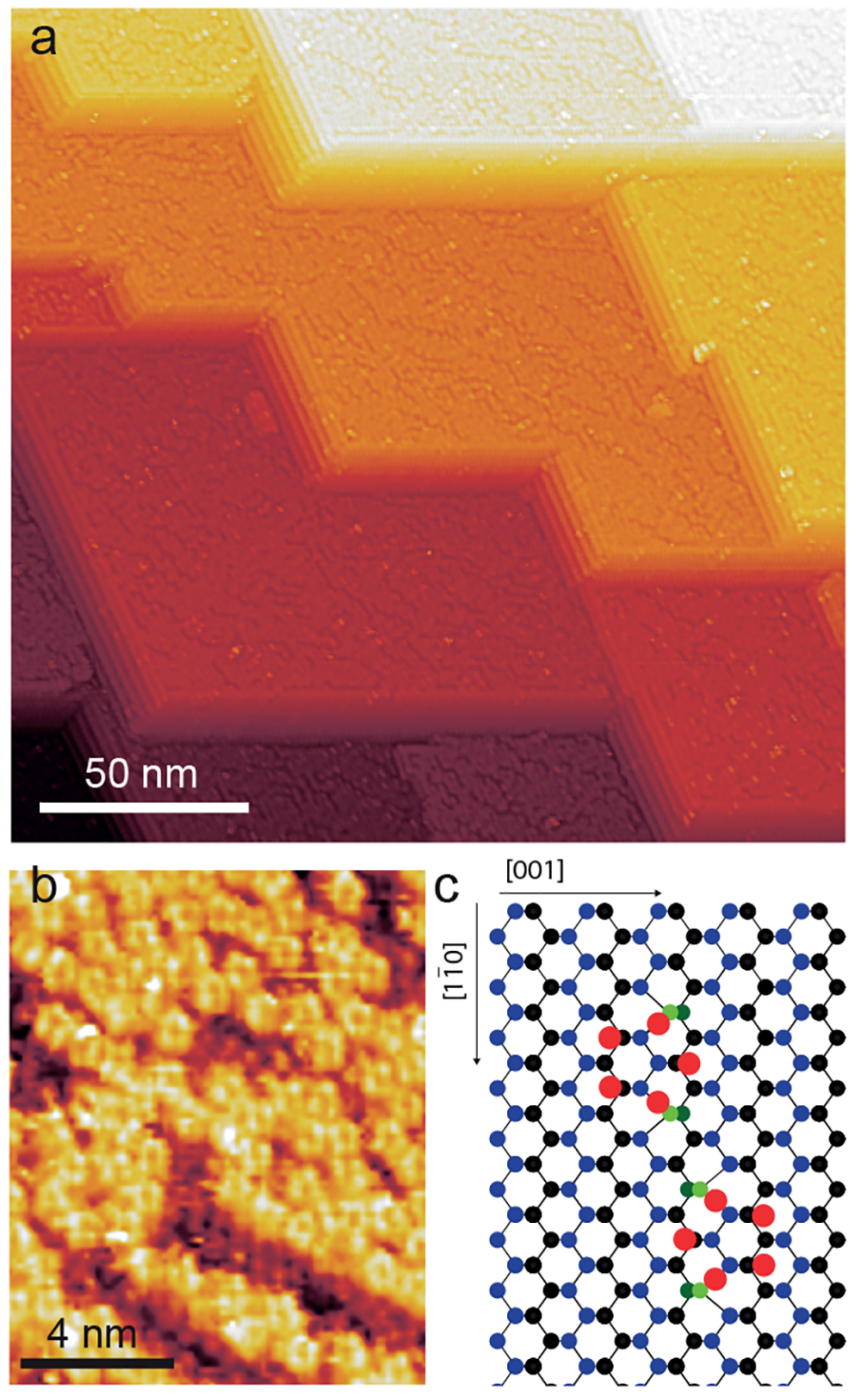
(a) Large scale STM image of the Ge(110) surface, setpoints: (0.2 nA, −1.5 V). (b) Small scale STM image of the Ge(110) terrace revealing the Ge pentagon clusters. (c) Structural model of the lowest energy binding sites of the Ge pentagons on Ge(110) adapted from Ichikawa.^46^ Red balls represent the pentagon atoms, and blue and black balls the Ge(110) upper and lower terrace, respectively, bright and dark green balls are displaced atoms of the upper and lower terrace by the presence of the Ge pentagons.

The clean Ge(110) crystal, Figure 1a, has large terraces separated by straight steps with heights corresponding to multiples of monoatomic layers. The Ge(110) surface is an intrinsically anisotropic surface. It has a higher surface free energy per unit area than the low-index (001) and (111) surfaces and thus tends to facet and reconstruct.^8,44,45^ The building block of the Ge(110) reconstructed surface is the Ge pentagon, a five-membered Ge ring. A random assembly of Ge pentagons is shown in the small-scale STM image of Figure 1b. The bulk-truncated Ge(110) surface has a rectangular symmetry that is composed of zigzag atomic rows running along the [11̅0] direction. The lower energy binding sites of Ge pentagons on the Ge(110) surface were calculated by Ichikawa^46^ and are adapted here, see Figure 1c. To accommodate the Ge pentagons, the underlying Ge–Ge bonds near the bridge sites are locally displaced in the [001] and [001̅] directions.^46^ In the relaxed configuration, shown in Figure 1c, a large lateral displacement of Ge atoms takes place in the layer under the pentagons with a root mean square displacement of 0.74 Å and 0.41 Å in the upper and the lower terrace, respectively.^46^

**Figure 2:**
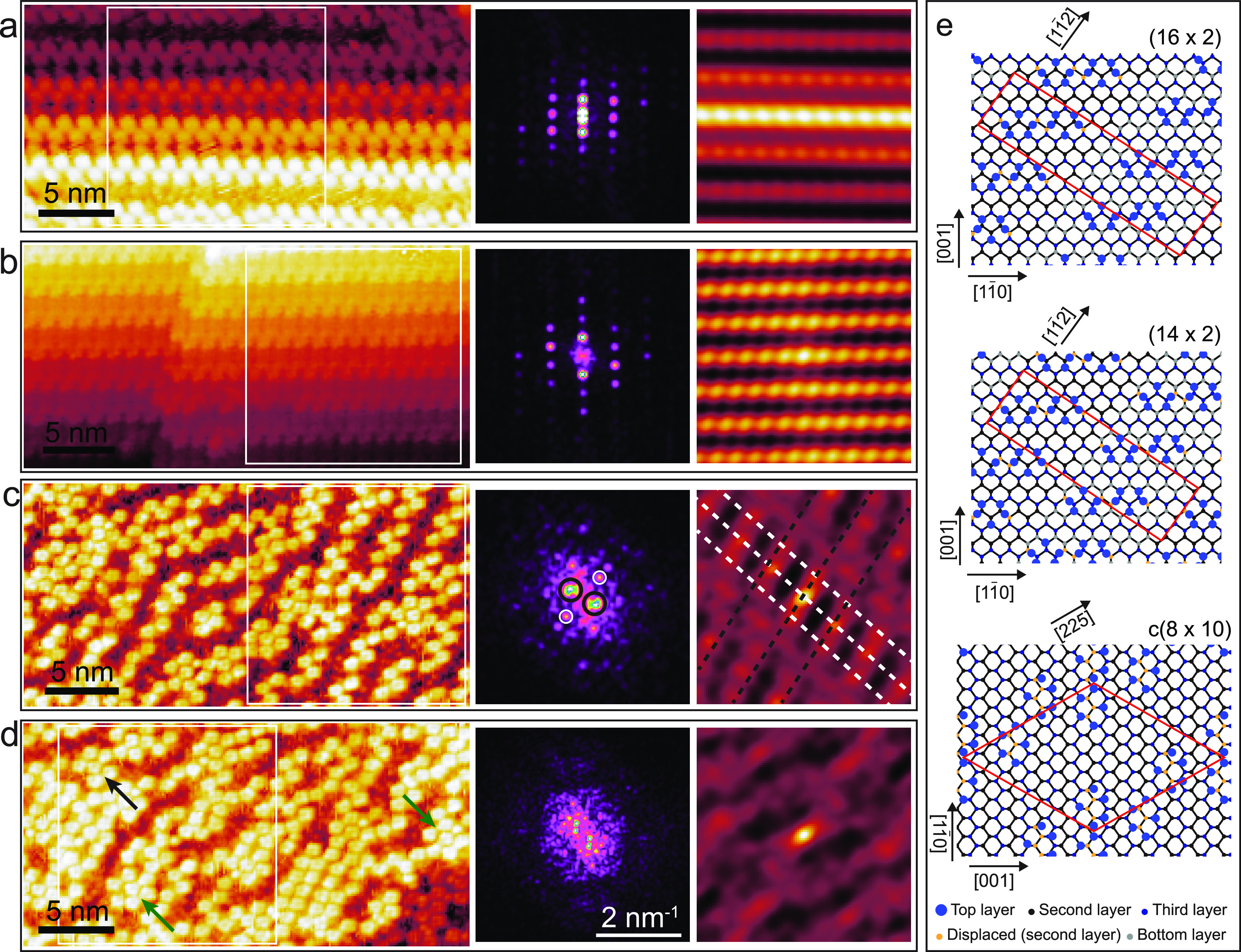
(a–d) Topographies (left images) and the corresponding fast Fourier transforms (middle panels) and self-correlation images (right panels) of the (16 × 2), (14 × 2), c(8 × 10), and disordered phases, respectively. (e) Structural models of the (16 × 2)-top, (14 × 2)-middle, and c(8 × 10)-top reconstructions. The models for the (16 × 2)-top and c(8 × 10) reconstructions are adapted from ref 46 and the (14 × 2) follows the symmetry of (16 × 2) but the nanofacets are one unit cell shorter.

The germanium pentagons form pairs and arrange themselves in a zig-zag fashion to form various reconstructions, namely, the (16 × 2) reconstruction, see Figure 2a, the newly discovered (14 × 2) reconstruction, see Figure 2b, the c(8 × 10) reconstruction, see Figure 2c, and a disordered phase, see Figure 2d. The corresponding fast Fourier transforms (FFTs) and self-correlation images (acquired similarly to refs (47) and (48)) of the topographies of each aforementioned reconstruction are given on the right side of panels (a) to (d). The (16 × 2) and (14 × 2) reconstructions, shown in Figure 2a,b, are the thermodynamically most stable reconstructions.^6,7^ They decorate step edges, resulting in a height difference between the two pentagon rows, which corresponds to the height of one atomic layer of the Ge(110) surface. The corresponding FFT images and self-correlation images in Figure 2a,b reveal the periodicities of the (16 × 2) and (14 × 2) reconstructions. From analyzing these images, we extract a row-to-row distance of 2.6 ± 0.05 and 2.3 ± 0.05 nm for the (16 × 2) and (14 × 2) reconstructions, respectively, and a periodicity along the rows corresponding to 1.4 nm ± 0.1 nm in both cases. As demonstrated in Figure 2a,b, the (14 × 2) forms on nanofacets that are a unit cell narrower than the (16 × 2) reconstruction. We note that we are the first to report on the (14 × 2) reconstruction. In addition to the (16 × 2) and (14 × 2) step reconstructions, the Ge(110) terraces contain two other distinct regions, namely, the c(8 × 10) surface reconstruction, Figure 2c, and disorder regions, Figure 2d. These pentagon-based reconstructions run on top of the Ge(110) bulk surface, along the [11̅0] direction. The structural models of the (16 × 2) and c(8 × 10) reconstructions, adapted from Ichikawa,^46^ are given in Figure 2e. The structural model of the (14 × 2) reconstruction follows the symmetry of the (16 × 2), but the nanofacets are a unit cell shorter as described above. In all cases, the reconstructions have large, highly complicated unit cells that involve multiple atomic layers.^5,8,46^

The FFT of the c(8 × 10) phase and the self-correlation image in Figure 2c reveal two prominent periodicities that correspond well with the expected ones for the c(8 × 10) structure. The diffuse background in the FFT is the result of the small coverage of this reconstruction. As indicated in the FFT image the middle bright spots (marked with black circles) correspond to the diagonal rows of pentagons along the [225] direction (see the structural model in Figure 2e for the c(8 × 10) structure). This periodicity is also visible in the self-correlation image, and it is marked with black dashed lines. The spots marked with the white circles in the FFT image and the white dashed stripes in the self-correlation image reflect the arrangement of pentagons along the [11̅1] direction. In the case of the disordered regions, Figure 2d, we obtain FFT images that are characterized by distinct shapes with the common appearance of a hexagon (similar, but not identical shapes have been observed in other disordered regions as well). Such a hexagonal order is also visible in the corresponding self-correlation image, right panel of Figure 2d. We attribute this quasi-hexagonal order to the tiling of the pentagons on the surface. As can be seen in the topography, densely packed regions tile in two frequent ways: (i) a pentagon surrounded by six pentagons (see green arrows in Figure 2d) and (ii) a pentagon surrounded by five pentagons (see black arrow in Figure 2d). The hexagonal arrangement appears to be the most frequent of the two and thus defines the observed pattern in both the FFT image and the self-correlation image. This reflects the limitations of the possible ways of ordering Ge pentagons on the surface. Taking such FFT and self-correlation patterns into consideration, we conclude that even in the “disordered” phase, the Ge pentagons cannot be completely random because of geometric constraints (there are only finite ways of tiling pentagons on a surface).

**Figure 3:**
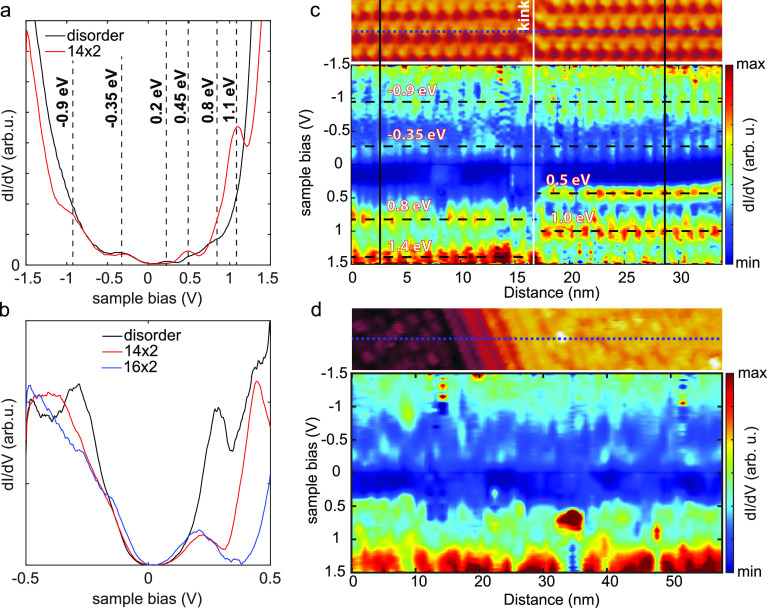
(a) Differential conductivity (d*I*/d*V*) versus the sample bias voltage (*V*) extracted from grid spectroscopy on top of a disordered region and on the (14 × 2) reconstruction. (b) d*I*/d*V*(*V*) spectra obtained on the (16 × 2), (14 × 2), and disordered reconstructions. (c) Top: STM image of the (14 × 2) reconstruction marking the location of the line spectroscopy. Bottom: d*I*/d*V*(*V*) measured along the blue striped line indicated in the top panel. Horizontal black dashed lines mark prominent states at positive and negative biases. Vertical white and dark-blue lines align the states to the exact topography. Setpoints: 0.5 nA, −1.5 V. (d) Top: STM topography of the Ge(110) surface. Bottom: d*I*/d*V*(*V*) line spectroscopy recorded along the line indicated in the top image.

The existence of both ordered and disordered phases composed of the same basic units (Ge pentagons) offers the unique possibility to understand the effect of geometric complexity on the local electronic structure of semiconductors. To undertake this challenge, we have employed scanning tunneling spectroscopy. We start our investigation by looking at differential conductance (d*I*(*V*)/d*V*) curves recorded on the disordered phase and comparing them to the ordered (14 × 2) reconstruction, see Figure 3a. The d*I*/d*V* curves in Figure 3a were obtained by averaging more than hundreds of single-point d*I*(*V*)/d*V* spectra, recorded in a grid fashion on the corresponding positions on the surface.

The differential conductance of the terraces and the (14 × 2) phase displayed similar electronic features. The (14 × 2) reconstruction has prominent electronic states at approximately −0.9, −0.35, 0.2, 0.45, 0.8, and 1.1 eV, respectively. The same states also appear on the terraces, albeit with slightly different intensities and energy positions. For example, the 0.45 eV state is much weaker in the disorder regions compared to the (14 × 2) reconstruction, and the −0.9 and 0.8 eV states appear only as shoulders. The differences in the spectra near the Fermi level (*E*_F_) are further amplified in the small voltage range d*I*(*V*)/d*V* spectra in Figure 3b. These differences in the DOS suggest that the exact arrangement of the Ge pentagons plays a significant role in the electronic structure of the surface. This poses the question of how the DOS changes locally. Dependence of the DOS on the pentagon arrangement is clear when looking at the LDOS of the (16 × 2) reconstruction, which is structurally very similar to the (14 × 2), but there are differences in the DOS near 0.45 eV, see Figure 3b. In the following, we demonstrate that these surface states have a localized character and originate either from the Ge pentagons or the underlying Ge–Ge back bonds and related modifications.

To reduce the complexity of the system, we start our investigation with the ordered (14 × 2) reconstruction, on which we have performed line spectroscopy along the blue dashed line in Figure 3c. The d*I*/d*V*(*V*) line spectroscopy is depicted in the bottom panel of Figure 3c. The d*I*/d*V*(*V*) curves along the line are recorded on top of the pentagons at the lower terrace (left side of the image) and in between the pentagons on the upper terrace (right side of the image). This phase shift is caused by the kink in the middle of the image. As shown in Figure 3c, there is a striking difference between the two regions, especially at voltages above the Fermi level. On the lower terrace (on top of the pentagons), states at −0.9, −0.35, 0.8, and 1.4 eV can be identified. Alignment of the exact line spectra with the corresponding topography in panel (c) reveals that the −0.9 and 1.4 eV are out of phase with the position of the pentagons (see vertical black line), indicating that the states originate from the underlying Ge–Ge bonds. On the other hand, the states at −0.35 and 0.8 eV align exactly with the position of the pentagons and can thus be linked to the Ge pentagons. This is also clear when looking at the right side of the image, where the −0.35 and 0.8 eV states are significantly weaker in intensity as a consequence of the tip measuring in between the pentagons. On this side, the state at −0.9 eV increases in intensity as expected. Two states are visible at 0.45 and 1.0 eV above the Fermi level. These states are not visible (or they are significantly lower in intensity) on the left side of the image, i.e., on the spectra acquired on top of the pentagons. Both states can therefore be linked to the Ge–Ge back bonds. Before looking at the disordered regions and spatial maps of the states, we will compare these results to earlier works on Si(110) and Ge(110) using angle-resolved photoemission spectroscopy (ARPES) and STS.^9,49^ Indeed similar states have been also identified on Si(110)–(16 × 2) with ARPES and STS.^9,49^ In the ARPES experiments,^9^ surface states located at −0.2, −0.4, −0.75, −1.0, and −1.5 eV have been identified. The state at −0.2 eV below the Fermi level was assigned to a pentagon state, while the other three states were assigned to Si edge states. Setvin et al.^49^ identified an additional state at +0.2 eV, which was attributed to a pentagon state. In the case of Ge(110)–c(8 × 10), photoemission experiments revealed a state at −1 eV, which was attributed to adatoms or rest-atoms.^6^ In contrast, we find states at −0.35, −0.9,+0.2,+0.45,+0.8,+1.1, and +1.5 eV. The surface states at −0.35 and 0.8 eV can be linked to pentagons, the states at −0.9, 0.45, and 1.1 eV to Ge–Ge back bonds, and the state at 0.2 eV to defects. As we will show next with high-resolution scanning tunneling spectroscopy, the states exist both in the ordered reconstructions and the disordered phases, albeit with differences in the exact energy position and intensity.

As shown in Figure 3d, the same states are also visible in the line spectroscopy acquired on the disordered terrace. In contrast to the ordered (14 × 2), the states on the disordered regions are varying seemingly randomly along the spectrum, reflecting the random arrangement of the pentagons and their influence on the Ge(110). Moreover, both in the (14 × 2) and disordered pentagon phases, there is a less pronounced state about 0.2 eV above the Fermi level. This state is very localized in both regions and is ascribed to a defect-induced state, caused either by a vacancy atom or an ad-atom.

**Figure 4:**
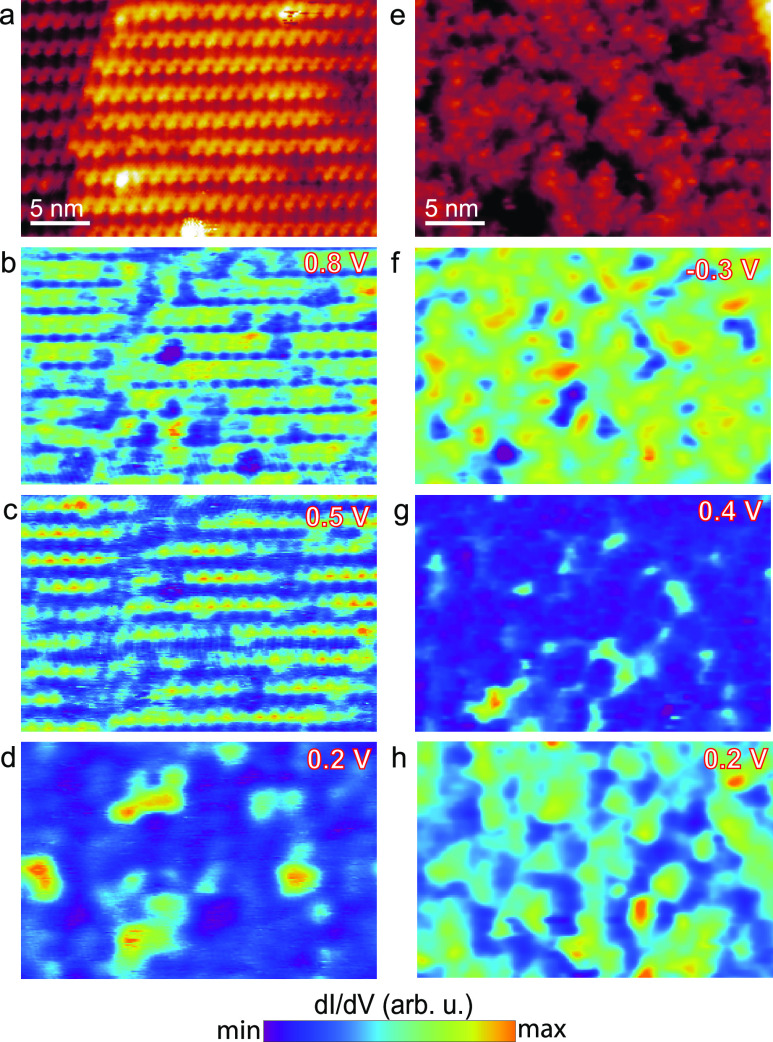
(a) STM image of the (14 × 2) reconstruction. (b–d) dI/dV maps recorded at the same region as in (a) for sample biases of 0.8 V (b), 0.5 V (c), and 0.2 V (d), corresponding to a pentagon state, a Ge–Ge back bond state, and a defect state, respectively (*I* = 0.5 nA). (e) STM topography image of a disordered pentagon region. (f–h) Corresponding d*I*/d*V* maps were recorded at the same area as in panel (e) for voltages −0.3 V (f), 0.4 V (g), and 0.2 V(h), corresponding to a pentagon, a Ge–Ge back bond, and a defect state, respectively.

The above observations can be confirmed by directly mapping the states using d*I*/d*V* maps at sample biases corresponding to the energies of the local states. Figure 4a shows the topography of the (14 × 2) phase. Figure 4b–d shows the corresponding d*I*/d*V* maps at energies of 0.8, 0.5, and 0.2 eV, respectively. In the case of 0.8 eV, panel (b), the d*I*/d*V* signal is the highest atop of the pentagons. This is inverted for a sample bias of 0.5 eV, where the narrow bright lanes correspond exactly to the areas between the zigzag rows, which represent the Ge–Ge back bonds. This is in line with Figure 3 and the assignment of the 0.8 eV peak to a pentagon state and the 0.5 eV to a back bond state. Both panels (b) and (c) follow the (14 × 2) symmetry albeit with inverted contrast in the case of panel (c). However, the d*I*/d*V* map at 0.2 eV, see Figure 4d, shows random bright features that do not follow the surface reconstruction. We thus ascribe them to surface defects randomly distributed over the surface. We did the same analysis on the disordered phase of Figure 4e. The corresponding d*I*/d*V* maps at energies of −0.3, 0.4, and 0.2 eV are shown in Figure 4f–h. Similar to the (14 × 2) reconstruction, the d*I*/d*V* is bright at the locations of pentagons at the pentagon state (−0.3 eV, see Figure 4f) and dim at 0.4 eV, Figure 4g (the Ge–Ge back bond state). The bright features at the 0.2 eV map in Figure 4h do not follow the surface morphology, suggesting that we are dealing with defect states.

**Figure 5:**
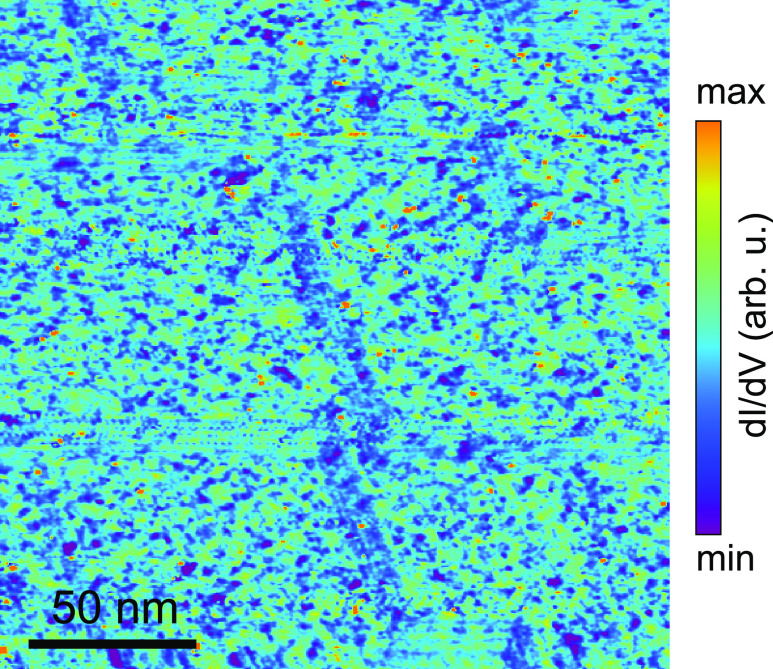
Large-scale (200 nm × 200 nm) d*I*/d*V* map recorded at −0.6 V of the Ge(110) surface. The image shows that the electronic disorder impacts the surface over large length scales.

We demonstrated that the way the Ge pentagons assembly on Ge(110) leads to spatial variations in the local density of states in the low-energy spectrum weight. As shown here, the Ge(110) surface serves as an excellent template to investigate and understand the influence of geometric constraints on electronic disorder using comprehensive scanning tunneling spectroscopy. As a final note, although the electronic disorder arises from geometric constraints and displacements on the atomic scale, it impacts the material across a larger length scale. This is shown in the large-s5cale d*I*/d*V* map in Figure 5, in which the same disordered electronic pattern runs across a surface of 200 × 200 nm^2^.

## Conclusions

We have used scanning tunneling microscopy and spectroscopy to investigate structural and electronic disorder in Ge(110). We have identified four surface reconstructions, of which three are ordered, i.e., (16 × 2), (14 × 2), and c(8 × 10), and one is disordered. Mapping of the local density of states of the ordered (14 × 2) reconstruction allowed us to pinpoint the exact origin of surface energy states to either surface pentagons or subsurface Ge–Ge bonds. The surface states at −0.35 and 0.8 eV can be linked to pentagon states, the energy states at −0.9, 0.45, and 1.1 eV to Ge–Ge back bond states and a state at 0.2 eV to a defect state. The states exist both in the ordered reconstructions and the disordered phase, albeit with differences in the exact energy position and intensity. They are spatially correlated with the position and order of the Ge pentagons, and even small changes in the locations of Ge pentagons cause significant changes in the spatial distribution and position of the surface states. This offers a wide configurational electronic landscape, which could open up new possibilities for data storage and computing applications.
